# Supplementary material for: Crystal structure and substrate-induced activation of ADAMTS13
Source: Nat Commun. 2019 Aug 22;10:3781. doi: 10.1038/s41467-019-11474-5 (PMC6706451; doi:10.1038/s41467-019-11474-5)
Supplement: Supplementary file 1 — Supplementary Information [file 41467_2019_11474_MOESM1_ESM.pdf]

Supplementary Information

Petri, Kim and Xu *et al*

"*Crystal structure and substrate-induced activation of ADAMTS13*"

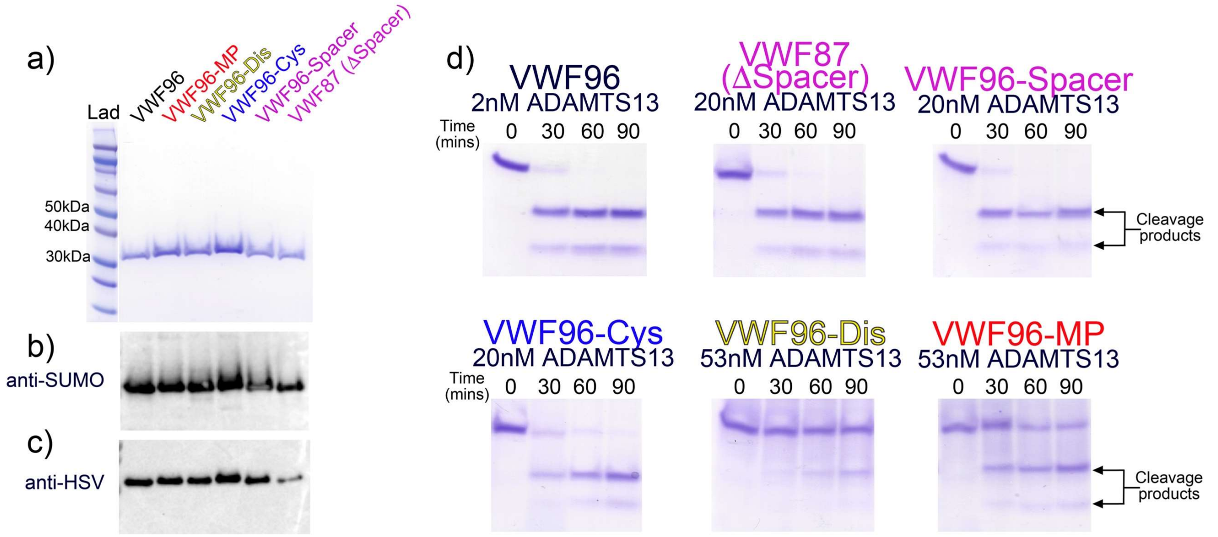

Supplementary Figure 1: Purification and proteolysis of VWF96 variants.
VWF96 and VWF96 variants were expressed in *E.coli* and purified to homogeneity by chromatography. **a)** VWF96 variants were analyzed by SDS-PAGE to assess purity. VWF96 is a 96 amino acid A2 domain fragment with a 13kDa N-terminal SUMO tag and a short C-terminal HSV tag and that spans the ADAMTS13 cleavage site and each of the Dis, Cys-rich and Spacer domain exosite binding regions. With N- and C-terminal tags, VWF96 has a MW of ~32kDa. VWF96 variants were also analyzed by Western blotting using either anti-SUMO (N-terminal tag) **(b)** or anti-HSV (C-terminal tag) **(c)** antibodies. **d)** Proteolysis of each VWF96 variant was analyzed qualitatively by incubation of 2nM – 53nM ADAMTS13 (concentration used for each substrate is given) with 3μM VWF96 variant. Sub-samples were taken at 0-90 mins, stopped with EDTA and analyzed by SDS-PAGE to visualize proteolysis. VWF96 was proteolyzed rapidly by 2nM ADAMTS13 with reactions approaching completion by ~30 mins. For VWF87(ΔSpacer), VWF96-Spacer and VWF96-Cys, 20nM ADAMTS13 was required to achieve a similar rate of proteolysis. For VWF96-Dis and VWF96-MP, 53nM ADAMTS13 was used. However, under these conditions only partial proteolysis was detected after 90 mins. Original gel and blot images are provided in the Source Data file.

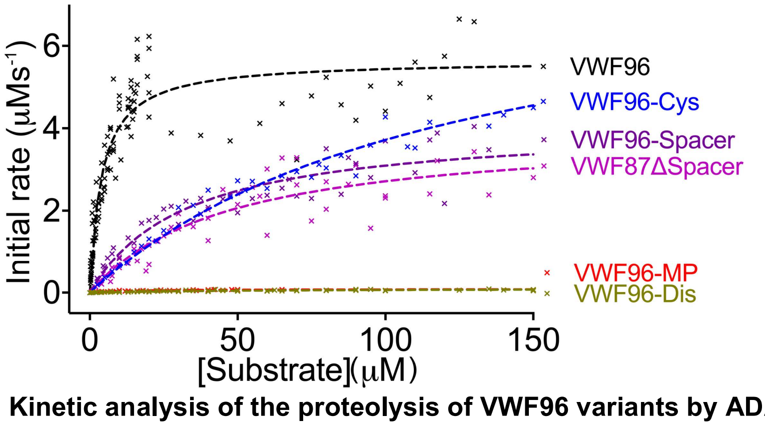

Supplementary Figure 2: Kinetic analysis of the proteolysis of VWF96 variants by ADAMTS13.
ADAMTS13 and VWF96, and variants thereof, were incubated at 37°C and proteolysis monitored over time by ELISA. From each progress curve, the initial rate of substrate proteolysis ADAMTS13 nM^{-1} was determined and plotted as a function of substrate concentration. Data were fitted to derive the V_{max} from which the k_{cat} was derived, and also the K_m (concentration of substrate at $V_{max}/2$). From these data, an independent determination of the catalytic efficiency (k_{cat}/K_m) for each substrate was derived – see Table 1. Data from reactions analyzing all variants (shown individually in Fig 3) are plotted on the same axis to enable their comparison. Note the markedly reduced k_{cat} for proteolysis of VWF96-MP and VWF96-Dis. Raw data underlying all reported averages are provided in the Source Data file corresponding to the data from Figure 2g–l.

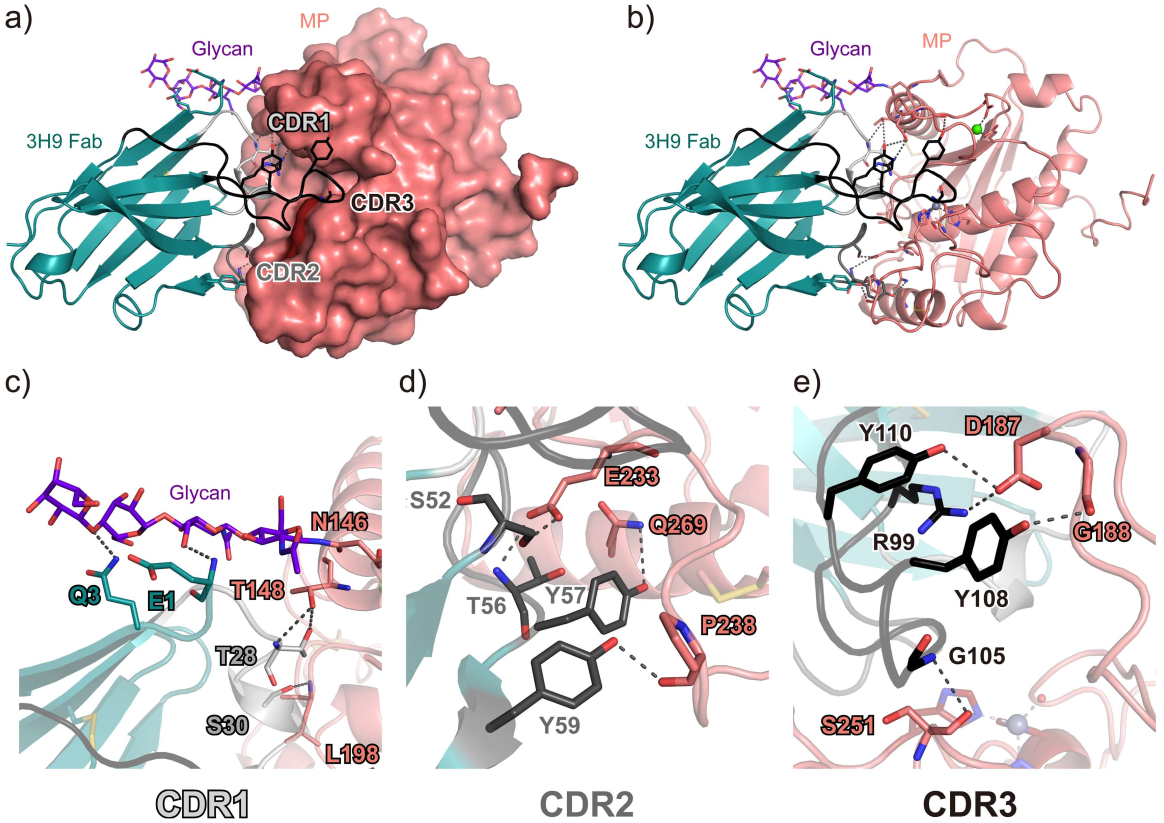

Supplementary Figure 3: Interaction of 3H9 Fab with ADAMTS13 MP domain.

a & b) Interface between the ADAMTS13 MP domain (light red) the 3H9 Fab heavy chain (teal). The three Fab CDRs are colored in grey as a ribbon (CDR1, residues 24-33), dark grey (CDR2, residues 52-58), and black (CDR3, residues 98-117) and the MP domain represented as surface (A) and ribbon (B). Zn^{2+} and Ca^{2+} ions are indicated as spheres. The glycan attached to Asn^{146} is shown in purple. **c-e)** Close up view of the interface between the ADAMTS13 MP domain and the Fab CDR1 (C), CDR2 (D) and CDR3 (E) loops with key interfacial residues shown as sticks. The principal electrostatic contacts between the 3H9 Fab and the MP domain are formed by electrostatic interactions between CDR2 and CDR3 and the protruding sidechains of two solvent exposed acidic residues Asp^{187} (CDR3) and Glu^{233} (CDR2) that flank the Zn^{2+} ion in the active-site. The Fab CDR3 loop forms the most extensive interaction through Arg^{99}, which forms a salt bridge with MP domain Asp^{187}. Fab residues Tyr^{108} and Tyr^{110} sidechain hydroxyl groups hydrogen bond to the Ca^{2+}-binding loop residues Asp^{187} and Gly^{188}, respectively, in the MP domain. Further contacts are made by CDR3 residue Asp^{103} packing against the MP domain His^{234} sidechain and the Trp^{102} sidechain packs against the peptide bond of Gly^{236} (not shown). The CDR2 loop interacts via Ser^{52} and Thr^{56} sidechains with the MP domain variable loop (residue 231-263) residue Glu^{233}. CDR2 residues Tyr^{59} and Tyr^{57} sidechains form interactions with MDTCS Pro^{238} and Arg^{268}/Gln^{269} respectively. CDR1 loop interacts with the MP domain Ca^{2+}-binding loop 180-193 and loop 231-263, respectively. Other interactions include the well-ordered saccharide moieties of the N-linked glycan attached to MP domain Asn^{146} hydrogen bonding to the Fab N-terminal residue Glu^{1}, the sidechain of Gln^{3} and the main chain carbonyl of the CDR1 loop residue Gly^{26} (not shown). Adjacent to this CDR1 residue Thr^{28} interacts with Ca^{2+}-binding loop residue Thr^{148} from MP domain $\alpha 1$ helix. Residues near the N-terminus of the Fab heavy chain and a single hydrogen bonding contact is formed between the Fab light chain residue Ser^{56} sidechain and the carbonyl tip of Asp^{187} in the Ca^{2+}-binding loop (not shown). Collectively these interactions cover the active-site, consistent with the inhibitory nature of the parent 3H9 mAb.

Supplementary Tables

	Fab-MDTCS<br>PDB ID: 6QIG
**Data collection**	
Space group	$P3_221$
Cell dimensions	
a, b, c (Å)	87.53, 87.53, 407.34
α, β, γ (°)	90, 90, 120
Resolution (Å)	46.2-2.8 (2.87-2.80)
R_{merge} (%)	16.7 (75.5)
$I / \sigma I$	11.7 (3.5)
Completeness (%)	99.3 (97.6)
Redundancy	12.5 (11.5)
**Refinement**	
Resolution (Å)	2.8 *
No. Reflections	45,840
R_{work} / R_{free} (%)	20.55 / 24.72
No. atoms	
Protein	7,797
Sugar	208
Metal	4
Water	183
B-factors (Å)	
Protein	50.27
Metal	62.23
Water	43.14
R.m.s. deviations	62.47
Bond lengths (Å)	0.010
Bond angles (°)	1.974

Supplementary Table 1: Crystallographic data collection and refinement statistics. * number of 3H9 Fab-MDTCS crystals analyzed = 4. Values in parentheses are for highest-resolution shell.

Primer pair name	Primer 1 seq	Primer 2 seq
VWF96	GGCAACAGGACCAACACTGGG	TCACCTCTGCAGCACCAGGTCAGG
VWF96-XhoI	TAATACGACTCACTATAGGG	TATACTCGAGCCTCTGCAGCACCAGGTCAG
VWF87-XhoI	TAATACGACTCACTATAGGG	TATACTCGAGTCGGGGGAGCCTCTCAAA
VWF96-MP	AATGTCTACATGGTCACCGGAAATCC	GTTGGGCGCCTGCTCCCGG
VWF96-Dis	GAGCTGCCTGGAACCATCCAGGTGGTGCCCATTGGAG	CTCCTGCTCAGCAGAGGCAGGATTTCCGGTGACC
VWF96-Cys	TCCCCTCAGCAGCAGCAGGACTTTGAGACGCTCCCC	ATTGGGATAGCCCTGCCTCTCCAGCTC
VWF96-Spacer	AATCAGCAGAGGCTCGAGATCAAACG	CGTGTCAGGAGCCTCTCGGGG
ADAMTS13 E225Q	GGAGTCACCATTGCCCATCAGATTGGGCACAGCTTCGGC	GCCGAAGCTGTGCCCAATCTGATGGGCAATGGTGACTCC

Supplementary Table 2: Primers used for amplification/generation of VWF96 variants. VWF96 was first amplified using the VWF96 primers and cloned into pET-SUMO. Thereafter, VWF96 and VWF87 (with the N-terminal SUMO tag were amplified using VWF96-XhoI or VWF87-XhoI and cloned into pET25b(+). From the resulting vectors, reverse PCR was performed to generate VWF96-MP, VWF96-Dis, VWF96-Cys and VWF96-Spacer using the respective primers. Blunt end PCR products were recircularized using T4 DNA ligase prior to transformation. The ADAMTS13 E225Q primer pair are those used for site-directed mutagenesis to substitute the active site Glu in ADAMTS13 to create and inactive enzyme.
