# Supplementary material for: Crystal structure and substrate-induced activation of ADAMTS13
Source: Nat Commun. 2019 Aug 22;10:3781. doi: 10.1038/s41467-019-11474-5 (PMC6706451; doi:10.1038/s41467-019-11474-5)
Supplement: Supplementary file 3 — Description of Additional Supplementary Files [file 41467_2019_11474_MOESM3_ESM.pdf]

Description of Additional Supplementary Files

File name: Supplementary Movie 1

Description: Movie showing the difference between the MDTCS structure (PDB: 6QIG) and the previous DTCS structure (3GHM). The movie starts showing the DTCS structure and morphs into our structure of MDTCS with the MP domain. Note the changes in the conformation of the TSP1 repeat (green) that causes the Dis domain (yellow) to approach the Cys-rich domain (blue).

File name: Supplementary Movie 2

Description: Movie showing the MP-Dis domains of ADAMTS13. Ribbon structure of the MP-Dis domains. MP domain is shown in light red, Dis domain in yellow. The active-site Zn^{2+} and bound Ca^{2+} ions are shown as spheres. The active-site and Dis domain exosite are highlighted in red. The 'gatekeeper triad' residues, Arg^{193}, Asp^{217} and Asp^{252} that interact ionically with each other are shown in grey. As the structure moves, note that the 'gatekeeper triad' residues block the active-site cleft occluding the passage between the Dis domain exosite and the active-site conferring enzyme latency. The 'gatekeep triad' interaction must be disrupted through engagement of the Dis exosite with VWF that induces an allosteric change in the MP domain to enable accommodation of a peptide substrate into the active-site cleft.
