# Supplementary material for: Crystal structure and substrate-induced activation of ADAMTS13
Source: Nat Commun. 2019 Aug 22;10:3781. doi: 10.1038/s41467-019-11474-5 (PMC6706451; doi:10.1038/s41467-019-11474-5)
Supplement: Supplementary file 6 — Reporting Summary [file 41467_2019_11474_MOESM6_ESM.pdf]

nature research

Corresponding author(s): James Crawley & Jonas Emsley

Last updated by author(s): Mar 5, 2019

Reporting Summary

Nature Research wishes to improve the reproducibility of the work that we publish. This form provides structure for consistency and transparency in reporting. For further information on Nature Research policies, see Authors & Referees and the Editorial Policy Checklist.

Statistics

For all statistical analyses, confirm that the following items are present in the figure legend, table legend, main text, or Methods section.

n/a	Confirmed	
☐	☒	The exact sample size (*n*) for each experimental group/condition, given as a discrete number and unit of measurement
☐	☒	A statement on whether measurements were taken from distinct samples or whether the same sample was measured repeatedly
☒	☐	The statistical test(s) used AND whether they are one- or two-sided<br>*Only common tests should be described solely by name; describe more complex techniques in the Methods section.*
☒	☐	A description of all covariates tested
☒	☐	A description of any assumptions or corrections, such as tests of normality and adjustment for multiple comparisons
☐	☒	A full description of the statistical parameters including central tendency (e.g. means) or other basic estimates (e.g. regression coefficient) AND variation (e.g. standard deviation) or associated estimates of uncertainty (e.g. confidence intervals)
☒	☐	For null hypothesis testing, the test statistic (e.g. *F*, *t*, *r*) with confidence intervals, effect sizes, degrees of freedom and *P* value noted<br>*Give P values as exact values whenever suitable.*
☒	☐	For Bayesian analysis, information on the choice of priors and Markov chain Monte Carlo settings
☒	☐	For hierarchical and complex designs, identification of the appropriate level for tests and full reporting of outcomes
☒	☐	Estimates of effect sizes (e.g. Cohen's *d*, Pearson's *r*), indicating how they were calculated

Our web collection on statistics for biologists contains articles on many of the points above.

Software and code

Policy information about availability of computer code

Data collection	Diffraction data were collected at I24, Diamond, using standard cryo mode (SPINE pins) on detector DECTRIS PILATUS3 S 6M (DECTRIS Ltd. Switzerland). XDSGUI (ver 25 Jan 2018, MPI for Medical Research, Heidelberg) was used for data reduction and ccp4i 7.0 (STFC Rutherford Appleton Laboratory) was used for scaling, phasing, and refinement.
Data analysis	Kinetic data were analyzed using GraphPad (Prism) v8<br>PDB files were analyzed and measurements made using PyMol 2.0 (Schrodinger LLC)

For manuscripts utilizing custom algorithms or software that are central to the research but not yet described in published literature, software must be made available to editors/reviewers. We strongly encourage code deposition in a community repository (e.g. GitHub). See the Nature Research guidelines for submitting code & software for further information.

Data

Policy information about availability of data

All manuscripts must include a data availability statement. This statement should provide the following information, where applicable:

- Accession codes, unique identifiers, or web links for publicly available datasets
- A list of figures that have associated raw data
- A description of any restrictions on data availability

Data supporting the findings of the study are available from the corresponding author upon reasonable request. Protein coordinates and structure factors have been deposited in the RCSB Protein Data Bank under code 6QIG.

October 2018

Field-specific reporting

Please select the one below that is the best fit for your research. If you are not sure, read the appropriate sections before making your selection.

☒ Life sciences ☐ Behavioural & social sciences ☐ Ecological, evolutionary & environmental sciences

For a reference copy of the document with all sections, see nature.com/documents/nr-reporting-summary-flat.pdf

Life sciences study design

All studies must disclose on these points even when the disclosure is negative.

Sample size	Sample sizes are most pertinent to the number of replicates used to derive kinetic data for the analysis of VWF96 and its variants by ADAMTS13. The sample sizes were determined based on our previous studies. For Michaelis-Menten kinetics, we collected n=27 to n=113 data sets (i.e. different substrate concentrations analyzed) to enable accurate fitting of the data and derivation of kinetic constants (kcat and Km). This appreciably exceeds the number of different datasets normally acquired for such studies, which often rely on 8-10 different concentrations. For time course kinetics, were repeated proteolysis assays n=3 to n=16, with each time point for each assay analyzed in triplicate.
Data exclusions	There were no data exclusions
Replication	The crystal structure was resolved from 4 different crystals.<br>For analysis of the kinetics of proteolysis of VWF96 and VWF96 variants, multiple preparations of both VWF96 and variants were analyzed using different preparations of ADAMTS13. For every batch/preparation of ADAMTS13, the specific activity of the preparation was measured. Any batch that exhibited reduced specific activity was not used for kinetic studies.
Randomization	Samples were not randomized as there was no aspect of this study for which randomization was relevant
Blinding	The investigators were not blinded to the identity of the different groups. The only aspect that this may have been pertinent to was the analysis of the proteolysis of VWF96 and its variants by ADAMTS13. However, as the conditions used (i.e. ADAMTS13 enzyme concentration and substrate concentration ranges) required knowledge of the VWF96 variant being studied, blinding was not possible.

Reporting for specific materials, systems and methods

We require information from authors about some types of materials, experimental systems and methods used in many studies. Here, indicate whether each material, system or method listed is relevant to your study. If you are not sure if a list item applies to your research, read the appropriate section before selecting a response.

Materials & experimental systems

n/a	Involved in the study	
☐	☒	Antibodies
☐	☒	Eukaryotic cell lines
☒	☐	Palaeontology
☒	☐	Animals and other organisms
☒	☐	Human research participants
☒	☐	Clinical data

Methods

n/a	Involved in the study	
☒	☐	ChIP-seq
☒	☐	Flow cytometry
☒	☐	MRI-based neuroimaging

Antibodies

Antibodies used	Mouse anti-ADAMTS13 mAb (3H9) was produced as a unique biological reagent in the lab of Prof K Vanhoorelbeke, KU Leuven, Belgium (coauthor)<br>Chicken anti-SUMO/SUMOstar pAb (LifeSensors) AB7002 LOT #: AB-41872.001<br>Goat anti-HSV (Bethyl) A190-136P LOT #: A190-136P-6
Validation	The 3H9 anti-ADAMTS13 MP domain mAb is a well-characterized mAb that inhibits ADAMTS13 by binding to the MP domain. This characterization has been reported in several published papers, and originally in Feys et al Blood 2011; 116(12):2005-2010<br>Chicken anti-SUMO/SUMOstar pAb (LifeSensors) AB7002 LOT #: AB-41872.001 was the only commercially available anti-SUMO antibody that recognized SUMO-tagged proteins specifically by ELISA. We analyzed four different antibodies for specificity by ELISA. For each preparation that we received, we tested the specificity of the antibody and compared its performance with previous aliquots. In all ELISAs, we included specific negative controls to test for non-specific binding/background, which in all cases was minimal. A blot is also provided in the Supp data demonstrating the specificity of the antibody for the SUMO-tagged proteins.<br>Goat anti-HSV (Bethyl) A190-136P was tested for its ability to recognize HSV-tagged proteins specifically by ELISA. For each preparation that we received, we tested the specificity of the antibody and compared its performance with previous aliquots. In

all ELISAs, we included specific negative controls to test for non-specific binding/background, which in all cases was minimal. A blot is also provided in the Supp data demonstrating the specificity of the antibody for the HSV-tagged proteins

Eukaryotic cell lines

Policy information about cell lines

Cell line source(s)	Drosophila Schneider 2 cells (ATCC); HEK293 cells (ATCC)
Authentication	No authentication was performed
Mycoplasma contamination	Mycoplasma contamination was not tested
Commonly misidentified lines (See ICLAC register)	The cell lines used are not commonly misidentified lines
